# Coccidioidal Placentitis With Normal Umbilical Artery Velocimetry

**DOI:** 10.1155/S1064744993000328

**Published:** 1993

**Authors:** Stephen A. Nickisch, Luis Izquierdo, Maggie A. Vill, Luis Curet, Gordon C. Wolf

**Affiliations:** 1 Divisions of Maternal Fetal Medicine and Reproductive Endocrinology Department of Obstetrics and Gynecology University of New Mexico School of Medicine Albuquerque NM USA; 2 Department of Obstetrics and Gynecology University of South Carolina Two Medical Park Columbia SC 29203 USA

## Abstract

*Background:* Disseminated coccidiomycosis during pregnancy can lead to both maternal and neonatal mortality. Placentitis is an uncommon sequelae and its effect on placental function remains speculative. The present report describes our management of such a case and describes serial umbilical artery velocimetry of an affected placenta.

*Case:* A pregnant woman with coccidioidal placentitis confirmed histologically was treated with systemic and intrathecal amphotericin B starting at 28 weeks gestation. Serial umbilical artery velocimetry revealed that all systolic/diastolic ratios remained normal, and a normal infant was delivered at term.

*Conclusion:* Coccidioidal placentitis was successfully treated with amphotericin B; serial umbilical artery velocimetry monitoring exhibited no abnormalities and, along with other reassuring fetal parameters, allowed continuation of the pregnancy to term.


Infectious Diseases in Obstetrics and Gynecology 1:144–148 (1993)

© 1993 Wiley-Liss, Inc.

### Coccidioidal Placentitis With Normal Umbilical Artery Velocimetry

**Stephen A. Nickisch, Luis Izquierdo, Maggie A. Vill, Luis Curet, and Gordon C. Wolf**

*Divisions of Maternal Fetal Medicine and Reproductive Endocrinology, Department of Obstetrics and Gynecology, University of New Mexico School of Medicine, Albuquerque, NM*

ABSTRACT

***Background:*** **Disseminated coccidiomycosis during pregnancy can lead to both maternal and neonatal mortality. Placentitis is an uncommon sequelae and its effect on placental function remains speculative. The present report describes our management of such a case and describes serial umbilical artery velocimetry of an affected placenta.**

***Case:*** **A pregnant woman with coccidioidal placentitis confirmed histologically was treated with systemic and intrathecal amphotericin B starting at 28 weeks gestation. Serial umbilical artery velocimetry revealed that all systolic/diastolic ratios remained normal, and a normal infant was delivered at term.**

***Conclusion:*** **Coccidioidal placentitis was successfully treated with amphotericin B; serial umbilical artery velocimetry monitoring exhibited no abnormalities and, along with other reassuring fetal parameters, allowed continuation of the pregnancy to term.** © 1993 Wiley-Liss, Inc.

KEY WORDS
Coccidiomycosis, Doppler, S/D ratios, amphotericin, meningitis

Coccidiomycosis affects 1 in every 1,000 pregnancies in endemic areas of the southwestern United States[1]; disseminated disease with meningitis and placentitis is much less common, with fewer than 60 cases having been reported. The use of amphotericin B and modern neonatal care have significantly reduced the previously high maternal and neonatal mortality associated with this disease, but a perinatal mortality of 4–14% remains for disseminated disease in pregnant patients.[2,3] Placental abnormalities associated with coccidioidal placental invasion have been postulated as the pathologic mechanisms explaining the increased perinatal morbidity and mortality. We hypothesized that these lesions might lead to measurable placental resistance values inasmuch as the characteristic coccidioidal changes (infarction, necrosis, and fibrin deposition) are common to the spectrum of lesions and lead to reduced placental parenchymal perfusion and increased umbilical artery resistance.[4] We report umbilical artery velocimetry as an indicator of placental resistance in a patient with coccidioidal meningitis and histologically confirmed coccidioidal placentitis.

### CASE REPORT

A 27-year-old Hispanic female, $G_2P_0Ab_1$, presented at 24 weeks gestation with an upper respiratory illness. She lived in central New Mexico though she had visited southern Arizona 6 weeks previously. Over a period of 2 weeks, the patient developed nausea, vomiting, headaches, blurred vision, fever, and weight loss. She had developed skin lesions on her upper lip and on her nose. A

**Address correspondence/reprint requests to Dr. G.C. Wolf, Department of Obstetrics and Gynecology, University of South Carolina, Two Medical Park, Columbia, SC 29203.**

Received August 12, 1993
Accepted October 1, 1993

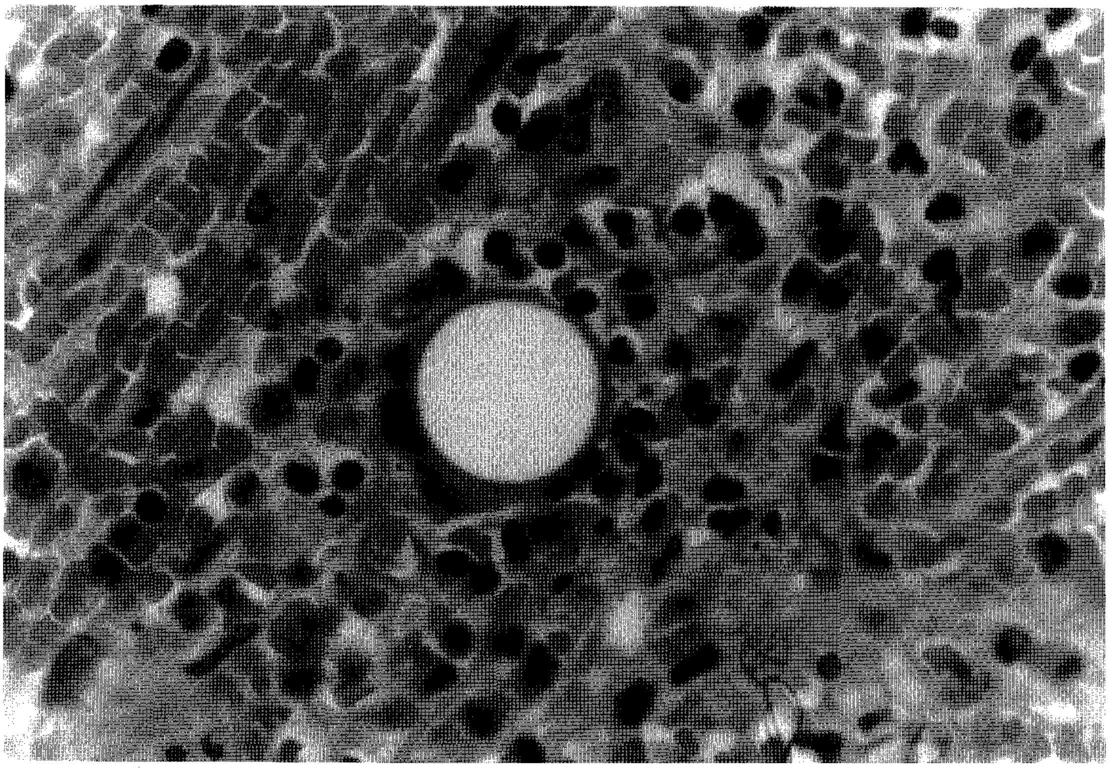

Fig. 1. Skin biopsy revealing spherule engulfed by multinucleated giant cells.

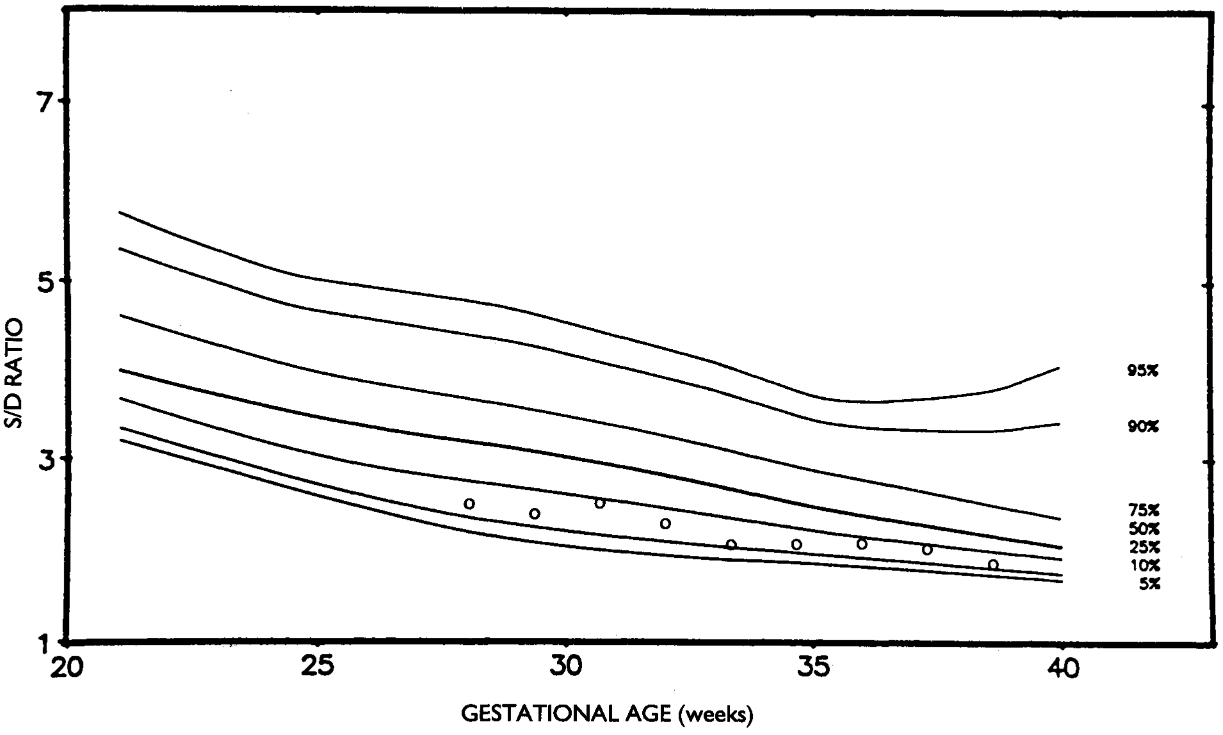

Fig. 2. Umbilical artery S/D ratios in the 10th to 25th percentile range.[4]

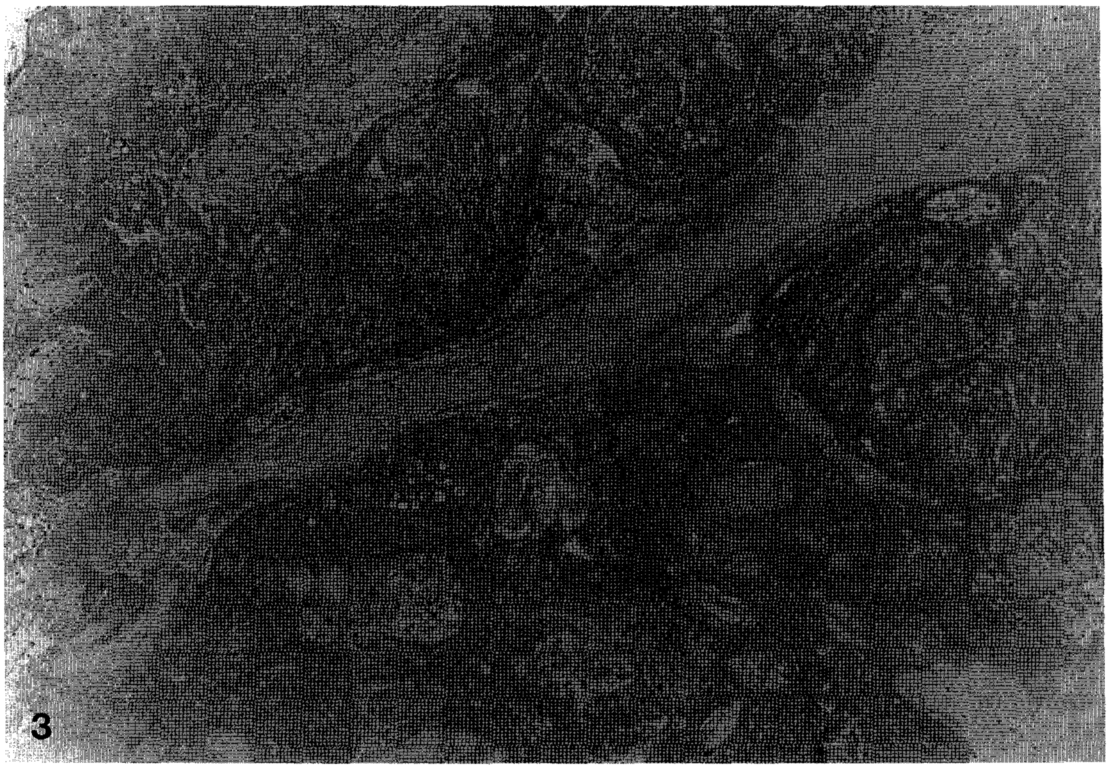

**Fig. 3. Placental biopsy confirming infarction necrosis and coccidioidal placentitis.**

biopsy confirmed *Coccidioides immitis* infection (Fig. 1). At 28 weeks, the coccidioidal complement fixation titer was found to be 1:8 in the cerebrospinal fluid and 1:64 in serum.

An ultrasound examination confirmed a 28-week male fetus, appropriate for gestational age. The umbilical artery systolic/diastolic (S/D) ratio determined at this time was in the 10th to 25th percentile at 2.5; ratios were subsequently determined at 3- to 7-day intervals (Fig. 2). Serial ultrasound examinations and nonstress tests likewise remained reassuring.

Treatment was initiated with systemic amphotericin B (to a maximum of 64 mg/day) in addition to intrathecal medication (0.3 mg on alternate days) until delivery at 38 weeks when the patient underwent spontaneous rupture of the fetal membranes. Oxytocin augmentation resulted in a normal spontaneous vaginal delivery. The infant weighed 3,130 g with APGAR scores of 9 and 10 at 1 and 5 minutes. The placenta appeared grossly normal except for three areas of infarction (confirmed microscopically), each smaller than 2 cm in diameter. Further microscopic examination revealed moderate amounts of intervillous fibrin deposition, numerous fungal spherules containing endospores and foreign-body giant cells, and an acute inflammatory reaction (Figs. 3, 4).

The patient's symptoms gradually resolved after the initiation of therapy, and the skin lesions diminished in size. Her total dose of amphotericin B at time of delivery had reached 4 g, and the regimen was changed to fluconazole, 400 mg daily, along with continuation of intracisternal amphotericin B, 0.5 mg weekly. The postpartum course was uneventful; she and the infant were discharged on the third day following delivery.

### DISCUSSION

The use of Doppler velocimetry has been described in the evaluation of many conditions in human pregnancy. Diabetes mellitus, systemic lupus erythematosus, hypertensive disorders of pregnancy, and sickle cell disease have all illustrated the utility of uterine-umbilical artery velocimetry in conditions associated with placental vascular disease.[5–8] However, Doppler investigation of pla-

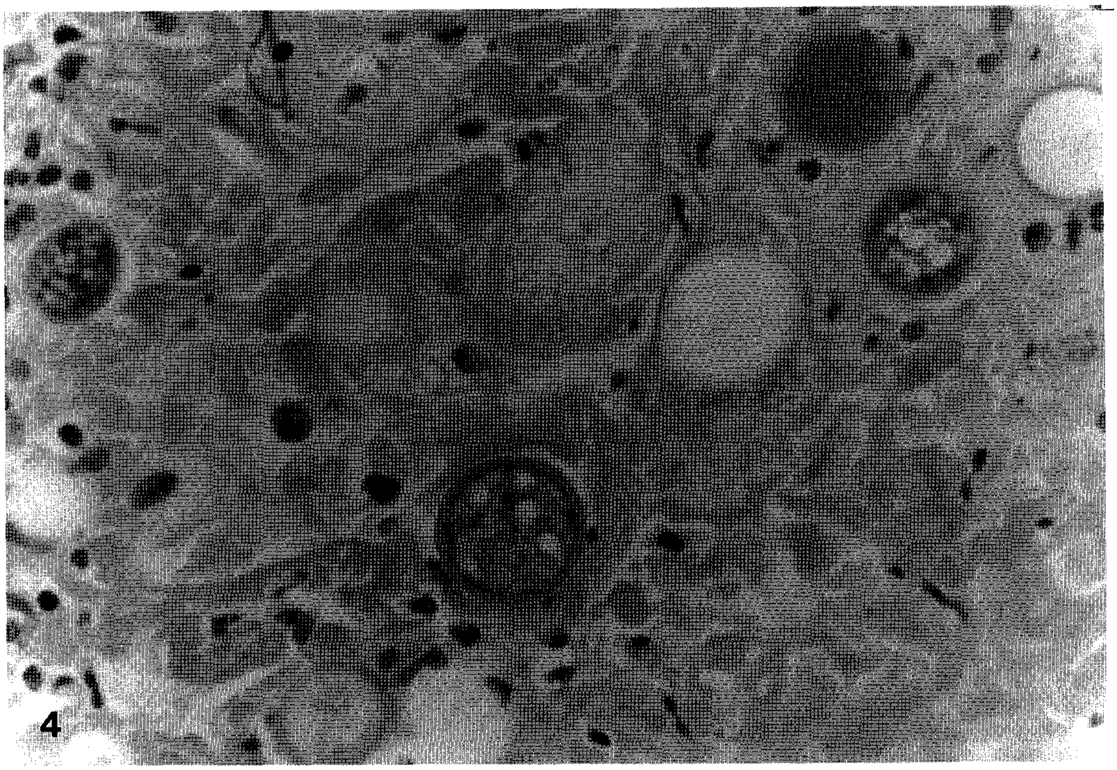

Fig. 4. Higher magnification of placenta with endospore-filled spherules.

centitis secondary to a specific infectious agent has not been reported.

A component of the pathophysiologic process in coccidiomycosis infection is that the hyphal outer wall of the fungal arthrocomidia of *C. immitis* resists phagocytosis, possibly as a result of the deposition of fibrinous material that inhibits polymorphonuclear cell access to the infectious agent.[1] The presence of placentitis with the concomitant fibrinous deposition and other pathologic features might lead to increased placental resistance and subsequent fetal compromise. Umbilical artery velocimetry serially throughout the third trimester, however, provided no evidence of functional placental compromise.

Despite the documentation of placental infection with the presence of multinucleated giant cells, fungal spherules and endospores, moderate intervillous fibrin deposition, and focal infarction with necrosis (Fig. 3), the umbilical artery measurements from 28 weeks to term showed no evidence of altered placental vascular resistance. The S/D ratio of the umbilical artery is plotted along the group of percentiles from Arabin's normative data (Fig. 4).[4]

The lack of correlation between pathologic findings and umbilical artery velocimetric data suggests that the functional effect of the placentitis in this case did not contribute to increased placental resistance and subsequent fetal compromise. It is possible that even a significant infectious process might not be reflected in fetal compromise unless there is a significant reduction in the number of terminal arteries in the tertiary villi.[9]

In summary, we report the birth of an appropriate-for-gestational-age infant at term without objective evidence of placental insufficiency despite disseminated maternal disease and significant coccidioidal placentitis. The lack of abnormal velocimetric measurements coupled with other reassuring fetal parameters may allow continued observation of preterm pregnancies affected by this and other infectious agents.

### REFERENCES

1. Peterson CM: Coccidiomycosis and pregnancy. Obstet Gynecol Surv 48:149–158, 1993.
2. Peterson CM, Johnson SL, Kelly JV, Kelly PC: Coccidioidal meningitis and pregnancy: A case report. Obstet Gynecol 23:835–836, 1989.

3. Walker MPR, Brody CZ, Resnik, R: Reactivation of coccidiomycosis in pregnancy. Obstet Gynecol 79:815–817, 1992.
4. Arabin B: Doppler Blood Flow Measurement in Uteroplacental and Fetal Vessels. New York: Springer-Verlag, pp 46, 61–75, 1990.
5. Bracero L, Schulman H, Fleischer A, Farmakides G, Rochelson B: Umbilical artery velocimetry in diabetes and pregnancy. Obstet Gynecol 68:654–657, 1986.
6. Ducy J, Schulman H, Farmakides G, Rochelson B, Bracero L, Fleischer A, Guzman E, Winter D, Penny B: A classification of hypertension in pregnancy based on Doppler velocimetry. Am J Obstet Gynecol 157:680–684, 1987.
7. Anyaegbunam A, Langer O, Brustman L, Damus K, Halpert R, Merkatz IR: The application of uterine and umbilical artery velocimetry to the antenatal supervision of pregnancies complicated by maternal sickle hemoglobinopathies. Am J Obstet Gynecol 159:544–547, 1988.
8. Guzman E: Uterine-umbilical artery Doppler velocimetry in pregnant women with systemic lupus erythematosus. J Ultrasound Med 11:275–281, 1982.
9. Trudinger BJ, Giles WB, Cook CM, Bombardieri J, Collins L: Fetal umbilical artery flow velocity waveforms and placental resistance: Clinical significance. Br J Obstet Gynaecol 92:23–30, 1985.
